# Intelligent mobile health management for the risk of gastrointestinal bleeding in anticoagulated patients with cardiovascular disease

**DOI:** 10.1590/1980-220X-REEUSP-2025-0311en

**Published:** 2026-06-15

**Authors:** Ting Li, Jingtao Zhang, Hua Li, Qinhu Zhang, Xiaoman Jiang, Jingjing Ma, Yan Feng

**Affiliations:** 1 Shaanxi People’s Hospital, Department of Cardiovascular Surgery, Xi’an, Shaanxi, China.; 2 Ankang Central Hospital, Department of Cardiology, Ankang, Shaanxi, China.; 3 Shaanxi People’s Hospital, Department of Gastroenterology, Xi’an, Shaanxi, China.

**Keywords:** Blood Coagulation, Cardiovascular Disease, Gastrointestinal Hemorrhage, Intelligence, Risk., Coagulação Sanguínea, Doenças Cardiovasculares, Hemorragia Gastrointestinal, Inteligência; Risco.

## Abstract

**Objective:**

To evaluate the effectiveness of a mobile health (mHealth)-based smart management strategy for reducing the risk of gastrointestinal bleeding in patients with cardiovascular disease undergoing anticoagulant therapy.

**Method:**

A retrospective observational study was conducted in a tertiary hospital, with patients undergoing anticoagulant therapy being allocated to conventional and smart groups. mHealth technology-based monitoring tools, standardized questionnaires for disease knowledge and self-assessment scales for anxiety and depression were used.

**Results:**

Related to hemostasis and satisfaction with the nursing staff were evaluated using t-tests and chi-square tests; p-values <0.05 were considered significant.

**Results:**

The mHealth-based smart management strategy showed a positive association with disease knowledge, a better psychological state, a shorter hemostasis time, and greater satisfaction with nursing care (P < 0.05).

**Conclusion:**

A smart management strategy based on mHealth was associated with better clinical and psychological outcomes than the conventional guidance group.

## RESUMO

**Objetivo:**

Avaliar a eficácia de uma estratégia de gestão inteligente baseada em saúde móvel (mHealth) para reduzir o risco de sangramento gastrointestinal em pacientes com doença cardiovascular submetidos a terapia anticoagulante.

**Método:**

Foi realizado um estudo observacional retrospectivo em um hospital terciário, no qual os pacientes submetidos a terapia anticoagulante foram alocados nos grupos convencional e inteligente. Foram utilizadas ferramentas de monitoramento baseadas em tecnologia mHealth, questionários padronizados para avaliação do conhecimento sobre a doença e escalas de autoavaliação para ansiedade e depressão. Os parâmetros relacionados à hemostasia e à satisfação com a equipe de enfermagem foram avaliados por meio de testes t e qui-quadrado; valores de p < 0,05 foram considerados significativos.

**Resultados:**

A estratégia de gestão inteligente baseada em mHealth mostrou uma associação positiva com o conhecimento da doença, um melhor estado psicológico, um tempo de hemostasia mais curto e maior satisfação com os cuidados de enfermagem (P < 0,05).

**Conclusão:**

Uma estratégia de gestão inteligente baseada em mHealth foi associada a melhores resultados clínicos e psicológicos do que o grupo de orientação convencional.

## INTRODUCTION

Cardiovascular diseases are common in clinical practice and are associated with high rates of morbidity, disability and mortality. Surgical intervention remains an important therapeutic approach for many patients. However, long-term anticoagulation therapy is often required after treatment to prevent thromboembolic events. While essential for reducing thrombotic risk in patients with cardiovascular disease, inappropriate use of anticoagulation therapy substantially increases the risk of bleeding complications, particularly gastrointestinal bleeding^(1)^. This creates a well-recognized clinical dilemma, whereby the benefits of anticoagulation must be carefully weighed against the risk of bleeding.

Gastrointestinal bleeding is one of the most frequent and severe complications associated with anticoagulation therapy, characterized by high mortality and rebleeding rates^(2,3)^. If not promptly identified and managed, it may lead to serious consequences including myocardial infarction, peripheral circulatory failure and sudden death, thereby posing a significant threat to patient safety^(4)^. Despite its clinical importance, conventional management strategies for anticoagulated patients are often limited by delayed risk warning, insufficient continuous monitoring and a lack of personalized interventions. Consequently, effective nursing management aimed at early risk warning and the prevention of bleeding complications plays a crucial role in improving the safety of anticoagulation therapy and optimizing clinical outcomes^(5,6)^.

In recent years, mobile health technologies (mHealth) have evolved rapidly, providing new opportunities to address challenges faced by patients on anticoagulation therapy. By integrating smartphones, wearable devices, telemedicine platforms and artificial intelligence algorithms, mHealth technologies enable real-time physiological monitoring, tracking of medication adherence, early warning of symptoms and individualized intervention^(7,8)^. Previous studies have demonstrated that nursing interventions delivered via a mobile platform can improve quality of life and nursing satisfaction among patients with cardiovascular disease^(9,10)^. However, these studies have largely focused on patient experience rather than bleeding risk prevention and safety management. Moreover, most patients with cardiovascular diseases are middle-aged or elderly individuals who have limited awareness of their condition and do not take their medication as prescribed, which increases their vulnerability to anticoagulant-related complications. To date, there has been insufficient systematic evaluation of mHealth-based strategies for assessing risk, providing early warnings and managing gastrointestinal bleeding in anticoagulated cardiovascular patients^(11,12)^.

Therefore, the present study aimed to evaluate the effectiveness of an intelligent MHealth-based risk management strategy for gastrointestinal bleeding in patients with cardiovascular disease undergoing anticoagulation therapy, focusing particularly on risk prevention, early intervention, and improving clinical safety.

## METHOD

### Study Design, Setting and Reporting Guideline

This was a single-center, retrospective, observational study conducted at Shaanxi People’s Hospital. The study period ran from February 2023 to February 2025.

It was designed and reported in accordance with the Strengthening the Reporting of Observational Studies in Epidemiology (STROBE) statement, as recommended by the EQUATOR Network.

### Ethical Aspects

The study was reviewed and approved by the Research Ethics Committee of Shaanxi People’s Hospital (approval number 2025K-498). Written informed consent was obtained from all participants prior to enrolment.

### Participants and Sampling Method

A total of 112 patients with cardiovascular disease who were receiving anticoagulation therapy were included in the study. Participants were recruited using a non-probabilistic convenience sampling method from eligible hospitalized patients during the study period. Following recruitment, all eligible patients received standard medical treatment and were then allocated to either the conventional group (n = 52) or the intelligent group (n = 60), according to the nursing strategy received. The conventional group comprised 29 males and 23 females aged 30–65 years (mean age 46.36 ± 4.26), of whom 18 had atrial fibrillation, nine had angina, 12 had myocardial infarction, five had pulmonary embolism and eight had other cardiovascular conditions. The intelligent group consisted of 35 men and 25 women aged 30–66 years (mean age 45.94 ± 5.31 years), including 21 cases of atrial fibrillation, 10 cases of angina, 15 cases of myocardial infarction, eight cases of pulmonary embolism and six other conditions. No significant differences in baseline sociodemographic or clinical characteristics were observed between the two groups (P > 0.05).

### Inclusion and Exclusion Criteria

The inclusion criteria were as follows: (1) patients who met the diagnostic criteria for gastrointestinal bleeding, as confirmed by gastroscopy and relevant clinical examinations^(13)^; (2) complete clinical and nursing records, obtained from electronic medical records; and (3) normal cognitive, auditory, reading and writing abilities, and the capacity to cooperate with study procedures.

Exclusion criteria included: Patients with upper gastrointestinal bleeding caused by other factors; patients complicated by hematological or infectious diseases; patients complicated by gastrointestinal tumors or other digestive system diseases; and patients taking immunosuppressants or gastric mucosal protective agents before admission.

### Group Assignment and Overall Workflow

After enrolment, patients were assigned to groups according to the nursing management strategy employed during their hospitalization. Nursing interventions were initiated during hospitalization and continued for four weeks after discharge.

### Conventional Nursing Care

Patients in the conventional group received routine nursing care, which included oxygen therapy, correction of electrolyte imbalances, monitoring of vital signs, guidance on medication, and discharge instructions.

### Smart Risk Management Based on Mobile Health/Monitoring via WeChat

The mobile application was developed independently by the hospital’s multidisciplinary clinical team, in collaboration with IT engineers. The team, which comprised cardiologists, gastroenterologists, pharmacists and senior nurses, formulated standardized dietary guidance protocols. The mobile system applied rule-based logic to provide reminders, and all recommendations were clinically reviewed rather than being generated by autonomous artificial intelligence algorithms. All patients in this group accessed the application via their own smartphones. Prior to the intervention, patients received standardized face-to-face training during hospitalization on how to use the application, operate the wearable device, report symptoms, and use the emergency alert function.

Post-discharge follow-up was conducted via WeChat. A designated nurse contacted each patient individually via private messaging once a week for four weeks after discharge. The follow-up included automated medication reminders, alerts about drug interactions, personalized dietary guidance and questions about symptoms such as melena, abdominal pain and dizziness. General health guidance was also provided through the mobile health platform. Dietary recommendations and food-drug interaction alerts were developed based on a combination of published clinical guidelines, literature evidence and expert consensus. Patients were also instructed to contact the nursing staff proactively via WeChat if they experienced new symptoms or an exacerbation of existing symptoms. Psychological support and health education were delivered via video modules and interactive question-and-answer functions, and patients with elevated anxiety or depression scores were referred to online counselling services. Interventions were initiated during hospitalization and continued throughout the four-week follow-up period. In addition, physiological indicators, including heart rate, blood pressure and blood oxygen saturation, were monitored using wearable devices. When predefined alert thresholds were reached, the system generated alerts requesting timely clinical attention. Multidisciplinary telemedicine support was also incorporated to facilitate dynamic risk assessment and clinical decision-making for higher-risk patients, involving cardiology, gastroenterology, and pharmacy teams.

### Outcome Measures and Data Collection Instruments

Clinical data were obtained from electronic medical and nursing records and supplemented by questionnaire- based assessments.

Hemostasis outcomes: Clinical efficacy was classified as markedly effective (stable vital signs, cessation of hematemesis and melena, and a negative fecal occult blood test), effective (hematemesis and melena in small volumes and a bleeding volume not exceeding 70 ml), or ineffective (a positive fecal occult blood test, no evident relief of melena and hematemesis, and a bleeding volume of at least 70 ml)^(14)^. The hemostasis response rate was calculated based on the following formula: Response rate = (number of markedly effective cases + number of effective cases)/total number of cases × 100%.

Risk of rebleeding: The risk of rebleeding was assessed before and after implementation (at discharge) using the Glasgow–Blatchford score (GBS), which is based on systolic blood pressure, urea, hemoglobin and clinical manifestations^(15)^. Scores of <6 and ≥6 correspond to low and intermediate-high risk, respectively.

Knowledge of the disease: A questionnaire designed specifically for this study was used to assess patients’ knowledge of the disease before and after the intervention. The questionnaire covered seven areas: knowledge of the disease; early identification of bleeding; self-care during bleeding; first aid measures; dietary guidelines; medication guidelines; and prevention of recurrences. Each section contained 20 questions, each scored from 1 to 5 points, for a total possible score of 100 points. Higher scores indicated greater knowledge of the disease. The internal consistency of the questionnaire was assessed using Cronbach’s α coefficient. The overall Cronbach’s α coefficient for the questionnaire was found to be 0.850 after analyzing the data from patients who participated in the research.

Psychological status: The Self-Rating Anxiety Scale (SAS) and Self-Rating Depression Scale (SDS) were used to assess patients’ psychological state before and after implementation (at discharge)^(16)^. Both the SAS and the SDS contained 20 items, each scored from 1 to 4 points. A higher score suggested more severe anxiety or depression. In the present study population, both scales demonstrated acceptable internal consistency reliability, with Cronbach’s α coefficients greater than 0.70, indicating good psychometric quality.

Nursing satisfaction: Patient satisfaction with nursing was evaluated using a satisfaction scale developed by the authors. This scale covered nursing time, the overall work capacity of nurses, nursing care, and prevention and healthcare. A total score of 100 points was possible, with scores of <55, 55–74, 75–90, and >90 corresponding to dissatisfied, generally satisfied, quite satisfied, and very satisfied, respectively. Satisfaction was calculated using the following formula: Total satisfaction = (number of highly satisfied cases + number of largely satisfied cases + number of generally satisfied cases)/total number of cases × 100%.

### Statistical Analysis

Statistical analysis was performed using SPSS 25.0 software. Measurement data that followed a normal distribution were described as mean ± standard deviation (*x* ± *s*) and compared between groups using an independent samples t-test. Count data were expressed as [n (%)] and subjected to the chi-squared test for comparison between groups. A statistically significant difference was denoted by P < 0.05.

## RESULTS

### Baseline Characteristics

A total of 112 patients were included in the analysis: 52 in the conventional group and 60 in the intelligent group. The baseline sociodemographic and clinical characteristics of the two groups are presented in Table 1. There were no statistically significant differences between the groups in terms of age, sex or major clinical characteristics (all P > 0.05), indicating good baseline comparability.

**Table 1:** Baseline sociodemographic and clinical characteristics of the study participants – X’an, Shaanxi Province, China, 2023.

Variable	Conventional group (n = 52)	Intelligent group (n = 60)	P value
Age (years)	46.36 ± 4.26	45.94 ± 5.31	0.464
Sex (male/female)	29/23	35/25	0.934
Atrial fibrillation, n (%)	18 (34.6)	21 (35.0)	0.875
Angina, n (%)	9 (17.3)	10 (16.7)	0.871
Myocardial infarction, n (%)	12 (23.1)	15 (25.0)	0.987
Pulmonary embolism, n (%)	5 (9.6)	8 (13.3)	0.751

### Hemostatic Effects

The hemostasis response rate was significantly higher in the intelligent group (98.33%) than in the conventional group (86.54%) (P < 0.05) (Table 2).

**Table 2:** Hemostatic effects [n (%)] – Xi’an, Shaanxi Province, China, 2023.

Group	n	Ineffective	Effective	Markedly effective	Response rate
Conventional	52	7 (13.46)	24 (46.15)	21 (40.38)	45 (86.54)
Intelligent	60	1 (1.67)	23 (38.33)	36 (60.00)	59 (98.33)
χ^2^					5.843
P					0.016

### Time to Hemostasis and Risk of Rebleeding

The time to hemostasis was significantly shorter in the intelligent group than in the conventional group (P < 0.05). There was no significant difference in the GBS score between the two groups before implementation (P > 0.05). However, after implementation, a significant decrease in the GBS score was observed in the intelligent group compared to the conventional group (P < 0.05) (see Table 3).

**Table 3:** Time to hemostasis and risk of rebleeding (x ± s) – Xi’an, Shaanxi Province, China, 2023.

Group	n	Time to hemostasis (h)	GBS (point)	
			Before implementation	After implementation
Conventional	52	32.15 ± 4.89	10.58 ± 2.10	7.97 ± 1.33^*^
Intelligent	60	23.59 ± 2.36	11.25 ± 2.49	6.11 ± 1.51^*^
*t*		12.043	1.526	6.868
P		<0.001	0.130	<0.001

P < 0.05 vs. this group before implementation.

### Disease Awareness

Following implementation, scores for disease awareness-related items increased in both groups compared to pre-implementation scores. These scores were higher in the intelligent group than in the conventional group (P < 0.05) (see Table 4).

**Table 4:** Disease awareness (x ± s) – Xi’an, Shaanxi Province, China, 2023.

Group	n	Disease knowledge		Early bleeding identification		Self-care during bleeding		First aid measures	
		Before implementation	After implementation	Before implementation	After implementation	Before implementation	After implementation	Before implementation	After implementation
Conventional	52	72.65 ± 7.83	88.63 ± 5.68^*^	74.22 ± 9.12	89.72 ± 5.49^*^	70.02 ± 8.70	89.71 ± 4.85^*^	71.62 ± 8.16	90.35 ± 4.82^*^
Intelligent	60	73.15 ± 7.73	94.06 ± 4.02^*^	72.38 ± 9.60	92.15 ± 4.85^*^	72.16 ± 7.72	93.50 ± 4.29^*^	71.16 ± 8.26	94.23 ± 3.68^*^
*t*		0.339	5.896	1.035	2.487	1.379	4.389	0.296	4.822
P		0.735	<0.001	0.303	0.014	0.171	<0.001	0.768	<0.001
**Group**	**n**	**Dietary guidelines**			**Medication guidelines**			**Relapse prevention**	
		**Before implementation**	**After implementation**		**Before implementation**	**After implementation**		**Before implementation**	**After implementation**
Conventional	52	77.61 ± 8.82	92.66 ± 3.63^*^		75.17 ± 6.40	91.21 ± 4.42^*^		73.22 ± 8.60	90.89 ± 4.00^*^
Intelligent	60	76.85 ± 7.54	95.50 ± 2.71^*^		76.07 ± 7.65	93.36 ± 3.81^*^		73.82 ± 8.02	93.83 ± 3.54^*^
*t*		0.492	4.729		0.669	2.765		0.382	4.127
P		0.624	<0.001		0.505	0.007		0.703	<0.001

P < 0.05 vs. this group before implementation.

### Psychological State

There were no significant differences in SDS and SAS scores between the two groups before implementation (P > 0.05). Following implementation, however, the intelligent group had significantly lower SDS and SAS scores than the conventional group (Table 5).

**Table 5:** Psychological state (x ± s) – Xi’an, Shaanxi Province, China, 2023.

Group	n	SDS score (point)		SAS score (point)	
		Before implementation	After implementation	Before implementation	After implementation
Conventional	52	61.35 ± 3.95	46.41 ± 2.44^*^	62.80 ± 5.74	46.30 ± 4.22^*^
Intelligent	60	62.23 ± 4.82	43.31 ± 2.25^*^	63.85 ± 5.61	41.45 ± 3.58^*^
*t*		1.047	6.992	0.977	6.581
P		0.298	<0.001	0.331	<0.001

P < 0.05 vs. this group before implementation.

### Satisfaction Degree

Satisfaction was significantly higher in the intelligent group than in the conventional group (95.00% *vs*. 76.92%, P < 0.05; see Table 6).

**Table 6:** Satisfaction degree [n (%)] – Xi’an, Shaanxi Province, China, 2023.

Group	n	Dissatisfied	Generally satisfied	Largely satisfied	Highly satisfied	Satisfaction
Conventional	52	12 (23.08)	18 (34.62)	12 (23.08)	10 (19.23)	40 (76.92)
Intelligent	60	3 (5.00)	15 (25.00)	20 (33.33)	22 (36.67)	57 (95.00)
χ^2^						7.848
P						0.005

## DISCUSSION

Anticoagulation therapy plays a key role in managing cardiovascular diseases such as atrial fibrillation and coronary heart disease. However, if anticoagulation is not managed correctly, the risk of bleeding complications increases substantially. Gastrointestinal bleeding is the most common anticoagulation-related bleeding event, accounting for around two-thirds of cases, and is associated with high mortality and rebleeding rates^(17,18)^. In the present study, patients receiving mHealth-based intelligent risk management demonstrated improved hemostasis outcomes and shorter times compared to those receiving conventional nursing care. These results suggest that the timely detection of bleeding-related symptoms and the early provision of nursing care, supported by mobile health technologies, play a critical role in stabilizing patients during anticoagulation therapy.

Additionally, the rebleeding risk assessed using GBS was more favorable in the intelligent management group at discharge. Previous studies have demonstrated that GBS is an effective tool for identifying patients at higher risk of adverse bleeding outcomes^(19,20)^. Our findings further suggest that integrating structured risk assessment with continuous nursing monitoring could improve clinical safety and reduce the risk of rebleeding in patients undergoing anticoagulation therapy.

Disease awareness is a key factor in determining treatment adherence and self-management in patients receiving long-term anticoagulation therapy^(21,22)^. This study showed that the intelligent group had significantly higher disease awareness scores than the conventional group. This indicates that mHealth-based intelligent risk management effectively improves patients’ understanding of gastrointestinal bleeding, medication use, dietary precautions and emergency responses. Educational video modules and interactive question-and-answer features on the mobile app helped patients to recognize symptoms early and reinforced correct self-care behaviors. These results are consistent with those of previous studies showing that mobile-based nursing education improves health literacy and self-management capacity in patients with chronic diseases^(23,24)^.

Previous literature has shown that psychological distress, including anxiety and depression, is prevalent among patients with cardiovascular diseases and is associated with poorer treatment adherence and worse clinical outcomes^(25,26)^. In line with previous studies^(27,28)^, our study showed that patients in the intelligent management group experienced significantly greater reductions in Self-Rating Anxiety Scale and Self-Rating Depression Scale scores than those receiving conventional care. Psychological support integrated into the mobile health platform, including timely counselling and peer support, may help alleviate emotional distress and encourage positive coping behaviors. Improved psychological status further enhances patients’ engagement in disease management, creating a positive feedback loop that supports hemostasis outcomes and overall nursing effectiveness^(29,30)^.

Nursing satisfaction reflects patients’ overall perceptions of the quality of care, communication and support they receive^(31)^. In the present study, satisfaction was significantly higher in the intelligent group than in the conventional group. This finding is consistent with previous evidence that mobile health interventions supported by technology improve patient-nurse interaction and responsiveness^(32)^. Continuous monitoring, personalized guidance and timely feedback may increase patients’ sense of security and trust, thereby enhancing satisfaction with nursing care.

## CONTRIBUTIONS TO NURSING PRACTICE

This study provides practical evidence that an intelligent risk management strategy based on mobile health technology can be effectively integrated into the nursing care of patients with cardiovascular disease who are receiving anticoagulation therapy. As well as improving patient outcomes, this tool supports and facilitates nursing professionals in delivering clinical care and health education. Combining hemostasis monitoring, rebleeding risk assessment, health education, psychological support and follow-up management promotes proactive, patient-centered nursing practice, enabling nurses to implement standardized, efficient, evidence-based care processes. The findings emphasize the potential of mobile health technologies to enhance nursing efficiency, alleviate the burden of repetitive risk assessment and educational tasks, and empower nurses to provide timely and consistent health education. Furthermore, the structured and user-friendly nature of this strategy may enhance nurses’ confidence and perceive effectiveness in anticoagulation management, thereby strengthening care quality and patient safety.

## LIMITATION OF THE STUDY

Several limitations should be acknowledged. Firstly, the study was conducted at a single center with a relatively small sample size, which may limit the generalizability of the findings. Secondly, the use of a non-probabilistic convenience sampling method may have introduced selection bias. Thirdly, the follow-up period was relatively short and did not allow for the full evaluation of the long-term effectiveness of the intelligent nursing intervention. Further validation of these findings requires future studies with larger sample sizes, multicenter designs and longer follow-up periods.

## CONCLUSION

The study demonstrated statistically significant improvements in hemostasis-related outcomes, disease awareness, psychological well-being and nursing satisfaction among anticoagulated patients with cardiovascular disease who received intelligent risk management via a mobile health platform. These findings suggest that this approach can contribute to safer and more effective anticoagulation management. Further studies on the subject are therefore needed, involving different populations and larger samples, taking a multicenter approach and conducting clinical trials, to establish healthcare strategies.

## Data Availability

The entire dataset supporting the results of this study is available upon request to the corresponding author.
