# Supplementary material for: Intelligent mobile health management for the risk of gastrointestinal bleeding in anticoagulated patients with cardiovascular disease
Source: Rev Esc Enferm USP. 2026 Jun 15;60:e20250311. doi: 10.1590/1980-220X-REEUSP-2025-0311en (PMC13274535; doi:10.1590/1980-220X-REEUSP-2025-0311en)
Supplement: Translation

# Translation: Gestión inteligente basada en salud móvil para el riesgo de hemorragia gastrointestinal en pacientes anticoagulados con enfermedad cardiovascular

**DOI:** 10.1590/1980-220X-REEUSP-2025-0311es

**Authors:** Ting Li, Jingtao Zhang, Hua Li, Qinhu Zhang, Xiaoman Jiang, Jingjing Ma, Yan Feng

**Affiliations:** 1 Shaanxi People’s Hospital, Department of Cardiovascular Surgery, Xi’an, Shaanxi, China.; 2 Ankang Central Hospital, Department of Cardiology, Ankang, Shaanxi, China.; 3 Shaanxi People’s Hospital, Department of Gastroenterology, Xi’an, Shaanxi, China.

**Keywords:** Coagulación Sanguínea, Enfermedades Cardiovasculares, Hemorragia Gastrointestinal, Inteligencia; Riesgo.

## Abstract

**Objetivo:**

Evaluar la eficacia de una estrategia de gestión inteligente basada en salud móvil (mHealth) para reducir el riesgo de hemorragia gastrointestinal en pacientes con enfermedad cardiovascular en tratamiento anticoagulante.

**Método:**

Se realizó un estudio observacional retrospectivo en un hospital terciario, en el que los pacientes en tratamiento anticoagulante fueron asignados a los grupos convencional e inteligente. Se utilizaron herramientas de monitoreo basadas en tecnología mHealth, cuestionarios estandarizados para evaluar el conocimiento sobre la enfermedad y escalas de autoevaluación de ansiedad y depresión. Los parámetros relacionados con la hemostasia y la satisfacción con el equipo de enfermería se analizaron mediante pruebas t y chi-cuadrado; se consideraron significativos valores de p < 0,05.

**Resultados:**

La estrategia de gestión inteligente basada en mHealth se asoció con mayor conocimiento sobre la enfermedad, mejor estado psicológico, menor tiempo de hemostasia y mayor satisfacción con los cuidados de enfermería (P < 0,05).

**Conclusión:**

La estrategia de gestión inteligente basada en mHealth se asoció con mejores resultados clínicos y psicológicos que la orientación convencional.

## INTRODUCCIÓN

Las enfermedades cardiovasculares son comunes en la práctica clínica y se asocian con altas tasas de morbilidad, discapacidad y mortalidad. La intervención quirúrgica sigue siendo un enfoque terapéutico importante para muchos pacientes. Sin embargo, a menudo se requiere un tratamiento anticoagulante a largo plazo después del tratamiento para prevenir eventos tromboembólicos. Si bien es esencial para reducir el riesgo trombótico en pacientes con enfermedad cardiovascular, el uso inadecuado del tratamiento anticoagulante aumenta sustancialmente el riesgo de complicaciones hemorrágicas, particularmente la hemorragia gastrointestinal^(1)^. Esto crea un dilema clínico bien reconocido, en el que los beneficios de la anticoagulación deben sopesarse cuidadosamente frente al riesgo de hemorragia.

La hemorragia gastrointestinal es una de las complicaciones más frecuentes y graves asociadas al tratamiento anticoagulante, caracterizada por altas tasas de mortalidad y de hemorragia recurrente^(2,3)^. Si no se identifica y se maneja con prontitud, puede tener consecuencias graves, como infarto de miocardio, insuficiencia circulatoria periférica y muerte súbita, lo que supone una amenaza significativa para la seguridad del paciente^(4)^. A pesar de su importancia clínica, las estrategias de manejo convencionales para los pacientes anticoagulados suelen verse limitadas por la alerta tardía de riesgos, el monitoreo continuo insuficiente y la falta de intervenciones personalizadas. En consecuencia, un manejo de enfermería eficaz, orientado a la alerta temprana de riesgos y a la prevención de complicaciones hemorrágicas, desempeña un papel crucial en la mejora de la seguridad del tratamiento anticoagulante y en la optimización de los resultados clínicos^(5,6)^.

En los últimos años, las tecnologías de salud móvil (mHealth) han evolucionado rápidamente, brindando nuevas oportunidades para abordar los desafíos que enfrentan los pacientes en tratamiento anticoagulante. Al integrar teléfonos inteligentes, dispositivos portátiles, plataformas de telemedicina y algoritmos de inteligencia artificial, las tecnologías de salud móvil permiten el monitoreo fisiológico en tiempo real, el seguimiento del cumplimiento de la medicación, la alerta temprana de síntomas y la intervención individualizada^(7,8)^. Estudios previos han demostrado que las intervenciones de enfermería prestadas a través de una plataforma móvil pueden mejorar la calidad de vida y la satisfacción con la atención de enfermería entre los pacientes con enfermedades cardiovasculares^(9,10)^. Sin embargo, estos estudios se han centrado en gran medida en la experiencia del paciente, más que en la prevención del riesgo de hemorragia y la gestión de la seguridad. Además, la mayoría de los pacientes con enfermedades cardiovasculares son personas de mediana edad o de la tercera edad que tienen un conocimiento limitado de su afección y no toman su medicación según lo prescrito, lo que aumenta su vulnerabilidad a las complicaciones relacionadas con los anticoagulantes. Hasta la fecha, no ha habido una evaluación sistemática suficiente de las estrategias basadas en la salud móvil (mHealth) para evaluar el riesgo, proporcionar alertas tempranas y manejar la hemorragia gastrointestinal en pacientes cardiovasculares anticoagulados^(11,12)^.

Por lo tanto, el presente estudio tuvo como objetivo evaluar la eficacia de una estrategia inteligente de manejo de riesgos basada en la salud móvil (mHealth) para la hemorragia gastrointestinal en pacientes con enfermedades cardiovasculares sometidos a terapia anticoagulante, centrándose particularmente en la prevención de riesgos, la intervención temprana y la mejora de la seguridad clínica.

## MÉTODO

### Diseño del Estudio, Entorno y Guía de Presentación de Informes

Este fue un estudio observacional, retrospectivo y de un solo centro, realizado en el Hospital Popular de Shaanxi. El período de estudio abarcó desde febrero de 2023 hasta febrero de 2025. Se diseñó y presentó de acuerdo con la declaración Strengthening the Reporting of Observational Studies in Epidemiology (STROBE), tal como lo recomienda la Red EQUATOR.

### Aspectos Éticos

El estudio fue revisado y aprobado por el Comité de Ética en Investigación del Hospital Popular de Shaanxi (número de aprobación 2025K-498). Se obtuvo el consentimiento informado por escrito de todos los participantes antes de su inclusión.

### Participantes y Método de Muestreo

Se incluyó en el estudio a un total de 112 pacientes con enfermedad cardiovascular que recibían tratamiento anticoagulante. Los participantes fueron reclutados mediante un método de muestreo por conveniencia no probabilístico a partir de pacientes hospitalizados elegibles durante el período de estudio. Tras el reclutamiento, todos los pacientes elegibles recibieron tratamiento médico estándar y luego fueron asignados al grupo convencional (n = 52) o al grupo inteligente (n = 60), según la estrategia de enfermería recibida. El grupo convencional estaba compuesto por 29 hombres y 23 mujeres de entre 30 y 65 años (edad media 46,36 ± 4,26), de los cuales 18 tenían fibrilación auricular, nueve tenían angina, 12 tenían infarto de miocardio, cinco tenían embolia pulmonar y ocho tenían otras afecciones cardiovasculares. El grupo inteligente estaba formado por 35 hombres y 25 mujeres de entre 30 y 66 años (edad media de 45,94 ± 5,31 años), incluyendo 21 casos de fibrilación auricular, 10 casos de angina de pecho, 15 casos de infarto de miocardio, ocho casos de embolia pulmonar y seis de otras afecciones. No se observaron diferencias significativas en las características sociodemográficas o clínicas basales entre los dos grupos (P > 0,05).

### Criterios de Inclusión y Exclusión

Los criterios de inclusión fueron los siguientes: (1) pacientes que cumplían los criterios diagnósticos de hemorragia gastrointestinal, confirmados mediante gastroscopia y exámenes clínicos pertinentes^(13)^; (2) registros clínicos y de enfermería completos, obtenidos de historias clínicas electrónicas; y (3) capacidades cognitivas, auditivas, de lectura y escritura normales, y capacidad para cooperar con los procedimientos del estudio.

Los criterios de exclusión incluyeron: pacientes con hemorragia gastrointestinal superior causada por otros factores; pacientes con complicaciones por enfermedades hematológicas o infecciosas; pacientes con complicaciones por tumores gastrointestinales u otras enfermedades del sistema digestivo; y pacientes que tomaban inmunosupresores o agentes protectores de la mucosa gástrica antes del ingreso.

### Asignación de Grupos y Flujo de Trabajo General

Tras la inscripción, se asignó a los pacientes a grupos según la estrategia de manejo de enfermería empleada durante su hospitalización. Las intervenciones de enfermería se iniciaron durante la hospitalización y continuaron durante cuatro semanas después del alta.

### Cuidados de Enfermería Convencionales

Los pacientes del grupo convencional recibieron cuidados de enfermería de rutina, que incluyeron oxigenoterapia, corrección de desequilibrios electrolíticos, monitoreo de signos vitales, orientación sobre medicación e instrucciones para el alta.

### Gestión Inteligente del Riesgo Basada en la Salud Móvil/Monitoreo a Través de WeChat

La aplicación móvil fue desarrollada de forma independiente por el equipo clínico multidisciplinario del hospital, en colaboración con ingenieros de TI. El equipo, compuesto por cardiólogos, gastroenterólogos, farmacéuticos y enfermeras de alto nivel, formuló protocolos estandarizados de orientación dietética. El sistema móvil aplicó una lógica basada en reglas para proporcionar recordatorios, y todas las recomendaciones fueron revisadas clínicamente en lugar de ser generadas por algoritmos autónomos de inteligencia artificial. Todos los pacientes de este grupo accedieron a la aplicación a través de sus propios teléfonos inteligentes. Antes de la intervención, los pacientes recibieron capacitación presencial estandarizada durante la hospitalización sobre cómo usar la aplicación, manejar el dispositivo portátil, informar síntomas y utilizar la función de alerta de emergencia.

El seguimiento posterior al alta se llevó a cabo a través de WeChat. Una enfermera designada se comunicó con cada paciente individualmente mediante mensajes privados una vez por semana durante cuatro semanas después del alta. El seguimiento incluyó recordatorios automáticos de medicación, alertas sobre interacciones farmacológicas, orientación dietética personalizada y preguntas sobre síntomas como melena, dolor abdominal y mareos. También se proporcionó orientación general sobre salud a través de la plataforma de salud móvil. Las recomendaciones dietéticas y las alertas de interacción entre alimentos y medicamentos se desarrollaron basándose en una combinación de guías clínicas publicadas, evidencia de la literatura y consenso de expertos. También se indicó a los pacientes que se pusieran en contacto con el personal de enfermería de manera proactiva a través de WeChat si experimentaban nuevos síntomas o una exacerbación de los síntomas existentes. El apoyo psicológico y la educación para la salud se proporcionaron a través de módulos de video y funciones interactivas de preguntas y respuestas, y los pacientes con puntuaciones elevadas de ansiedad o depresión fueron derivados a servicios de asesoramiento en línea. Las intervenciones se iniciaron durante la hospitalización y continuaron a lo largo del período de seguimiento de cuatro semanas. Además, se monitorearon indicadores fisiológicos, como la frecuencia cardíaca, la presión arterial y la saturación de oxígeno en sangre, utilizando dispositivos portátiles. Cuando se alcanzaban los umbrales de alerta predefinidos, el sistema generaba alertas solicitando atención clínica oportuna. También se incorporó apoyo multidisciplinario de telemedicina para facilitar la evaluación dinámica del riesgo y la toma de decisiones clínicas para los pacientes de mayor riesgo, con la participación de equipos de cardiología, gastroenterología y farmacia.

### Medidas de Resultado e Instrumentos para la Recolección de Datos

Los datos clínicos se obtuvieron de los registros médicos y de enfermería electrónicos y se complementaron con evaluaciones basadas en cuestionarios.

Resultados de hemostasia: La eficacia clínica se clasificó como marcadamente eficaz (signos vitales estables, cese de la hematemesis y la melena, y prueba de sangre oculta en heces negativa), eficaz (hematemesis y melena en pequeños volúmenes y un volumen de sangrado que no excedía los 70 ml) o ineficaz (prueba de sangre oculta en heces positiva, sin alivio evidente de la melena y la hematemesis, y un volumen de sangrado de al menos 70 ml)^(14)^. La tasa de respuesta hemostática se calculó según la siguiente fórmula: Tasa de respuesta = (número de casos marcadamente eficaces + número de casos eficaces)/número total de casos × 100 %.

Riesgo de hemorragia recurrente: El riesgo de hemorragia recurrente se evaluó antes y después de la intervención (al alta) utilizando la puntuación de Glasgow-Blatchford (GBS), que se basa en la presión arterial sistólica, la urea, la hemoglobina y las manifestaciones clínicas^(15)^. Las puntuaciones <6 y ≥6 corresponden a riesgo bajo y medio-alto, respectivamente.

Conocimiento de la enfermedad: Se utilizó un cuestionario diseñado específicamente para este estudio con el fin de evaluar el conocimiento de la enfermedad por parte de los pacientes antes y después de la intervención. El cuestionario abarcaba siete áreas: conocimiento de la enfermedad; identificación temprana de la hemorragia; autocuidado durante la hemorragia; medidas de primeros auxilios; pautas dietéticas; pautas de medicación; y prevención de recidivas. Cada sección contenía 20 preguntas, cada una puntuada de 1 a 5 puntos, para una puntuación total posible de 100 puntos. Las puntuaciones más altas indicaban un mayor conocimiento de la enfermedad. La consistencia interna del cuestionario se evaluó utilizando el coeficiente α de Cronbach. Se encontró que el coeficiente α de Cronbach general para el cuestionario era de 0,850 tras analizar los datos de los pacientes que participaron en la investigación.

Estado psicológico: Se utilizaron la Escala de Autoevaluación de la Ansiedad (Self-Rating Anxiety Scale – SAS) y la Escala de Autoevaluación de la Depresión (Self-Rating Depression Scale – SDS) para evaluar el estado psicológico de los pacientes antes y después de la implementación (al momento del alta)^(16)^. Tanto la SAS como la SDS contenían 20 ítems, cada uno puntuado de 1 a 4 puntos. Una puntuación más alta indicaba una ansiedad o depresión más grave. En la población del presente estudio, ambas escalas demostraron una confiabilidad de consistencia interna aceptable, con coeficientes α de Cronbach superiores a 0,70, lo que indica una buena calidad psicométrica.

Satisfacción con la atención de enfermería: La satisfacción de los pacientes con la atención de enfermería se evaluó utilizando una escala de satisfacción desarrollada por los autores. Esta escala abarcaba el tiempo de atención de enfermería, la capacidad laboral general de las enfermeras, los cuidados de enfermería y la prevención y la atención médica. La puntuación total posible era de 100 puntos, correspondiendo las puntuaciones <55, 55–74, 75–90 y >90 a insatisfecho, generalmente satisfecho, bastante satisfecho y muy satisfecho, respectivamente. La satisfacción se calculó utilizando la siguiente fórmula: Satisfacción total = (número de casos muy satisfechos + número de casos bastante satisfechos + número de casos generalmente satisfechos)/número total de casos × 100 %.

### Análisis Estadístico

El análisis estadístico se realizó con el software SPSS 25.0. Los datos de medida que seguían una distribución normal se describieron como media ± desviación estándar (*x* ± *s*) y se compararon entre los grupos mediante una prueba t para muestras independientes. Los datos de recuento se expresaron como [n (%)] y se sometieron a la prueba de chi-cuadrado para la comparación entre los grupos. Se consideró una diferencia estadísticamente significativa un valor de P < 0,05.

## RESULTADOS

### Características Basales

Se incluyó en el análisis a un total de 112 pacientes: 52 en el grupo convencional y 60 en el grupo inteligente. Las características sociodemográficas y clínicas basales de los dos grupos se presentan en la Tabla 1. No hubo diferencias estadísticamente significativas entre los grupos en cuanto a edad, sexo o características clínicas principales (todos P > 0,05), lo que indica una buena comparabilidad basal.

**Tabla 1:** Características sociodemográficas y clínicas basales de los participantes en el estudio – Xi’an, Shaanxi Province, China, 2023.

Variable	Grupo convencional (n = 52)	Grupo inteligente (n = 60)	Valor p
Edad (años)	46,36 ± 4,26	45,94 ± 5,31	0,464
Sexo (hombre/mujer)	29/23	35/25	0,934
Fibrilación auricular, n (%)	18 (34,6)	21 (35,0)	0,875
Angina, n (%)	9 (17,3)	10 (16,7)	0,871
Infarto de miocardio, n (%)	12 (23,1)	15 (25,0)	0,987
Embolia pulmonar, n (%)	5 (9,6)	8 (13,3)	0,751

### Características Basales

Se incluyó en el análisis a un total de 112 pacientes: 52 en el grupo convencional y 60 en el grupo inteligente. Las características sociodemográficas y clínicas basales de los dos grupos se presentan en la Tabla 1. No hubo diferencias estadísticamente significativas entre los grupos en cuanto a edad, sexo o características clínicas principales (todas p > 0,05), lo que indica una buena comparabilidad basal.

### Efectos Hemostáticos

La tasa de respuesta hemostática fue significativamente mayor en el grupo inteligente (98,33 %) que en el grupo convencional (86,54 %) (P < 0,05) (Tabla 2).

**Tabla 2:** Efectos hemostáticos [n (%)] – Xi’an, provincia de Shaanxi, China, 2023.

Grupo	n	Ineficaz	Eficaz	Notablemente eficaz	Tasa de respuesta
Convencional	52	7 (13,46)	24 (46,15)	21 (40,38)	45 (86,54)
Inteligente	60	1 (1,67)	23 (38,33)	36 (60,00)	59 (98,33)
χ^2^					5,843
P					0,016

### Tiempo Hasta la Hemostasia y Riesgo de Hemorragia Recurrente

El tiempo hasta la hemostasia fue significativamente más corto en el grupo inteligente que en el grupo convencional (P < 0,05). No hubo diferencias significativas en la puntuación GBS entre los dos grupos antes de la implementación (P > 0,05). Sin embargo, tras la implementación, se observó una disminución significativa de la puntuación GBS en el grupo inteligente en comparación con el grupo convencional (P < 0,05) (véase la Tabla 3).

**Tabla 3:** Tiempo hasta la hemostasia y riesgo de hemorragia recurrente (x ± s) – Xi’an, provincia de Shaanxi, China, 2023.

Grupo	n	Tiempo hasta la hemostasia (h)	GBS (puntos)	
			Antes de la implementación	Después de la implementación
Convencional	52	32,15 ± 4,89	10,58 ± 2,10	7,97 ± 1,33^*^
Inteligente	60	23,59 ± 2,36	11,25 ± 2,49	6,11 ± 1,51^*^
*t*		12,043	1,526	6,868
P		<0,001	0,130	<0,001

P < 0,05 *frente* a este grupo antes de la implementación.

### Concienciación Sobre la Enfermedad

Tras la implementación, las puntuaciones de los ítems relacionados con el conocimiento de la enfermedad aumentaron en ambos grupos en comparación con las puntuaciones previas a la implementación. Estas puntuaciones fueron más altas en el grupo inteligente que en el grupo convencional (P < 0,05) (véase la Tabla 4).

**Tabla 4:** Concienciación sobre la enfermedad (x ± s) – Xi’an, provincia de Shaanxi, China, 2023.

Grupo	n	Conocimiento de la enfermedad		Detección precoz de la hemorragia		Autocuidado durante la hemorragia		Medidas de primeros auxilios	
		Antes de la implementación	Después de la implementación	Antes de la implementación	Después de la implementación	Antes de la implementación	Después de la implementación	Antes de la implementación	Después de la implementación
Convencional	52	72,65 ± 7,83	88,63 ± 5,68^*^	74,22 ± 9,12	89,72 ± 5,49^*^	70,02 ± 8,70	89,71 ± 4,85^*^	71,62 ± 8,16	90,35 ± 4,82^*^
Inteligente	60	73,15 ± 7,73	94,06 ± 4,02^*^	72,38 ± 9,60	92,15 ± 4,85^*^	72,16 ± 7,72	93,50 ± 4,29^*^	71,16 ± 8,26	94,23 ± 3,68^*^
*t*		0,339	5,896	1,035	2,487	1,379	4,389	0,296	4,822
P		0,735	<0,001	0,303	0,014	0,171	<0,001	0,768	<0,001
**Grupo**	**n**	**Recomendaciones dietéticas**			**Pautas de medicación**			**Prevención de recaídas**	
		**Antes de la implementación**	**Después de la implementación**		**Antes de la implementación**	**Después de la implementación**		**Antes de la implementación**	**Después de la implementación**
Convencional	52	77,61 ± 8,82	92,66 ± 3,63^*^		75,17 ± 6,40	91,21 ± 4,42^*^		73,22 ± 8,60	90,89 ± 4,00^*^
Inteligente	60	76,85 ± 7,54	95,50 ± 2,71^*^		76,07 ± 7,65	93,36 ± 3,81^*^		73,82 ± 8,02	93,83 ± 3,54^*^
*t*		0,492	4,729		0,669	2,765		0,382	4,127
P		0,624	<0,001		0,505	0,007		0,703	<0,001

P < 0,05 *frente* a este grupo antes de la implementación.

### Estado Psicológico

No se observaron diferencias significativas en las puntuaciones del SDS y el SAS entre los dos grupos antes de la implementación (P > 0,05). Sin embargo, tras la implementación, el grupo inteligente presentó puntuaciones del SDS y el SAS significativamente más bajas que el grupo convencional (Tabla 5).

**Tabla 5:** Estado psicológico (x ± s) – Xi’an, provincia de Shaanxi, China, 2023.

Grupo	n	Puntuación SDS (puntos)		Puntuación SAS (puntos)	
		Antes de la implementación	Después de la implementación	Antes de la implementación	Después de la implementación
Convencional	52	61,35 ± 3,95	46,41 ± 2,44^*^	62,80 ± 5,74	46,30 ± 4,22^*^
Inteligente	60	62,23 ± 4,82	43,31 ± 2,25^*^	63,85 ± 5,61	41,45 ± 3,58^*^
*t*		1,047	6,992	0,977	6,581
P		0,298	<0,001	0,331	<0,001

P < 0,05 frente a este grupo antes de la implementación.

### Grado de Satisfacción

La satisfacción fue significativamente mayor en el grupo inteligente que en el grupo convencional (95,00 % frente a 76,92 %, P < 0,05; véase la Tabla 6).

**Tabla 6:** Grado de satisfacción [n (%)] – Xi’an, provincia de Shaanxi, China, 2023.

Grupo	n	Insatisfecho	Conforme en general	Muy satisfecho	Muy satisfecho	Satisfacción
Convencional	52	12 (23,08)	18 (34,62)	12 (23,08)	10 (19,23)	40 (76,92)
Inteligente	60	3 (5,00)	15 (25,00)	20 (33,33)	22 (36,67)	57 (95,00)
χ^2^						7,848
P						0,005

## DISCUSIÓN

El tratamiento anticoagulante desempeña un papel fundamental en el manejo de enfermedades cardiovasculares como la fibrilación auricular y la cardiopatía coronaria. Sin embargo, si la anticoagulación no se controla adecuadamente, el riesgo de complicaciones hemorrágicas aumenta considerablemente. La hemorragia gastrointestinal es el episodio hemorrágico más frecuente relacionado con la anticoagulación, ya que representa alrededor de dos tercios de los casos, y se asocia con altas tasas de mortalidad y de hemorragia recurrente^(17,18)^. En el presente estudio, los pacientes que recibieron una gestión inteligente del riesgo basada en mHealth mostraron mejores resultados hemostáticos y tiempos más cortos en comparación con los que recibieron cuidados de enfermería convencionales. Estos resultados sugieren que la detección oportuna de los síntomas relacionados con la hemorragia y la prestación temprana de cuidados de enfermería, con el apoyo de tecnologías de salud móvil, desempeñan un papel fundamental en la estabilización de los pacientes durante el tratamiento anticoagulante.

Además, el riesgo de hemorragia recurrente evaluado mediante el GBS fue más favorable en el grupo de manejo inteligente al momento del alta. Estudios previos han demostrado que el GBS es una herramienta eficaz para identificar a los pacientes con mayor riesgo de resultados adversos de hemorragia^(19,20)^. Nuestros hallazgos sugieren además que la integración de la evaluación estructurada del riesgo con el monitoreo continuo de enfermería podría mejorar la seguridad clínica y reducir el riesgo de hemorragia recurrente en pacientes sometidos a terapia anticoagulante.

La conciencia de la enfermedad es un factor clave para determinar la adherencia al tratamiento y el autocontrol en pacientes que reciben terapia anticoagulante a largo plazo^(21,22)^. Este estudio mostró que el grupo inteligente obtuvo puntuaciones de conciencia de la enfermedad significativamente más altas que el grupo convencional. Esto indica que la gestión inteligente del riesgo basada en mHealth mejora eficazmente la comprensión de los pacientes sobre la hemorragia gastrointestinal, el uso de medicamentos, las precauciones dietéticas y las respuestas de emergencia. Los módulos de video educativos y las funciones interactivas de preguntas y respuestas de la aplicación móvil ayudaron a los pacientes a reconocer los síntomas de manera temprana y reforzaron los comportamientos correctos de autocuidado. Estos resultados concuerdan con los de estudios previos que muestran que la educación de enfermería basada en dispositivos móviles mejora la alfabetización en salud y la capacidad de autocontrol en pacientes con enfermedades crónicas^(23,24)^.

La literatura previa ha demostrado que el malestar psicológico, incluyendo la ansiedad y la depresión, es frecuente entre los pacientes con enfermedades cardiovasculares y se asocia con una menor adherencia al tratamiento y peores resultados clínicos^(25,26)^. En consonancia con estudios previos^(27,28)^, nuestro estudio mostró que los pacientes del grupo de manejo inteligente experimentaron reducciones significativamente mayores en las puntuaciones de la Escala de Autoevaluación de la Ansiedad y la Escala de Autoevaluación de la Depresión que aquellos que recibieron atención convencional. El apoyo psicológico integrado en la plataforma de salud móvil, que incluye asesoramiento oportuno y apoyo entre pares, puede ayudar a aliviar el malestar emocional y fomentar comportamientos de afrontamiento positivos. La mejora del estado psicológico refuerza aún más la participación de los pacientes en el manejo de la enfermedad, creando un ciclo de retroalimentación positiva que favorece los resultados de la hemostasia y la eficacia general de la atención de enfermería^(29,30)^.

La satisfacción con la atención de enfermería refleja las percepciones generales de los pacientes sobre la calidad de la atención, la comunicación y el apoyo que reciben^(31)^. En el presente estudio, la satisfacción fue significativamente mayor en el grupo inteligente que en el grupo convencional. Este hallazgo concuerda con la evidencia previa de que las intervenciones de salud móvil respaldadas por la tecnología mejoran la interacción y la capacidad de respuesta entre el paciente y el personal de enfermería^(32)^. El monitoreo continuo, la orientación personalizada y la retroalimentación oportuna pueden aumentar la sensación de seguridad y confianza de los pacientes, mejorando así la satisfacción con la atención de enfermería.

## Contribuciones A la Práctica de Enfermería

Este estudio proporciona evidencia práctica de que una estrategia inteligente de gestión de riesgos basada en la tecnología de salud móvil puede integrarse eficazmente en la atención de enfermería de pacientes con enfermedades cardiovasculares que reciben terapia anticoagulante. Además de mejorar los resultados de los pacientes, esta herramienta apoya y facilita a los profesionales de enfermería la prestación de atención clínica y educación para la salud. La combinación de la monitorización de la hemostasia, la evaluación del riesgo de hemorragia recurrente, la educación sanitaria, el apoyo psicológico y la gestión del seguimiento promueve una práctica de enfermería proactiva y centrada en el paciente, lo que permite a las enfermeras implementar procesos de atención estandarizados, eficientes y basados en la evidencia. Los hallazgos enfatizan el potencial de las tecnologías de salud móvil para mejorar la eficiencia de la enfermería, aliviar la carga de las tareas repetitivas de evaluación de riesgos y educación, y empoderar a las enfermeras para que brinden educación sanitaria oportuna y consistente. Además, la naturaleza estructurada y fácil de usar de esta estrategia puede aumentar la confianza de las enfermeras y su percepción de la eficacia en el manejo de la anticoagulación, fortaleciendo así la calidad de la atención y la seguridad del paciente.

## LIMITACIONES DEL ESTUDIO

Se deben reconocer varias limitaciones. En primer lugar, el estudio se llevó a cabo en un solo centro con un tamaño de muestra relativamente pequeño, lo que puede limitar la generalización de los hallazgos. En segundo lugar, el uso de un método de muestreo por conveniencia no probabilístico puede haber introducido un sesgo de selección. En tercer lugar, el período de seguimiento fue relativamente corto y no permitió evaluar plenamente la eficacia a largo plazo de la intervención de enfermería inteligente. Para validar estos hallazgos se requieren estudios futuros con muestras más grandes, diseños multicéntricos y períodos de seguimiento más prolongados.

## CONCLUSIÓN

El estudio demostró mejoras estadísticamente significativas en los resultados relacionados con la hemostasia, la conciencia de la enfermedad, el bienestar psicológico y la satisfacción con la atención de enfermería entre los pacientes con enfermedades cardiovasculares en tratamiento anticoagulante que recibieron una gestión inteligente del riesgo a través de una plataforma de salud móvil. Estos hallazgos sugieren que este enfoque puede contribuir a una gestión de la anticoagulación más segura y eficaz. Por lo tanto, se necesitan más estudios sobre el tema, que incluyan diferentes poblaciones y muestras más grandes, adopten un enfoque multicéntrico y realicen ensayos clínicos, para establecer estrategias de atención de salud.

## Data Availability

El conjunto de datos que respaldan las conclusiones de este estudio está disponible previa solicitud al autor correspondiente
